# Epithelial mesenchymal transition (EMT): a universal process in lung diseases with implications for cystic fibrosis pathophysiology

**DOI:** 10.1186/s12931-018-0834-8

**Published:** 2018-07-18

**Authors:** Nathan Rout-Pitt, Nigel Farrow, David Parsons, Martin Donnelley

**Affiliations:** 1 Robinson Research Institute, University of Adelaide, Adelaide, South Australia Australia; 2 Adelaide Medical School, University of Adelaide, Adelaide, South Australia Australia; 3 Department of Respiratory and Sleep Medicine, Women’s and Children’s Hospital, 72 King William Rd, North Adelaide, South Australia 5006 Australia; 4 Australian Respiratory Epithelium Consortium (AusRec), Perth, Western Australia 6105 Australia

**Keywords:** Epithelial mesenchymal transition, Cystic fibrosis, Lung, Fibrosis, E-cadherin

## Abstract

Cystic Fibrosis (CF) is a genetic disorder that arises due to mutations in the Cystic Fibrosis Transmembrane Conductance Regulator gene, which encodes for a protein responsible for ion transport out of epithelial cells. This leads to a disruption in transepithelial Cl-, Na + and HCO_3_− ion transport and the subsequent dehydration of the airway epithelium, resulting in infection, inflammation and development of fibrotic tissue. Unlike in CF, fibrosis in other lung diseases including asthma, chronic obstructive pulmonary disease and idiopathic pulmonary fibrosis has been well characterised. One of the driving forces behind fibrosis is Epithelial Mesenchymal Transition (EMT), a process where epithelial cells lose epithelial proteins including E-Cadherin, which is responsible for tight junctions. The cell moves to a more mesenchymal phenotype as it gains mesenchymal markers such as N-Cadherin (providing the cells with migration potential), Vimentin and Fibronectin (proteins excreted to help form the extracellular matrix), and the fibroblast proliferation transcription factors Snail, Slug and Twist. This review paper explores the EMT process in a range of lung diseases, details the common links that these have to cystic fibrosis, and explores how understanding EMT in cystic fibrosis may open up novel methods of treating patients with cystic fibrosis.

## Background

Cystic Fibrosis (CF) is a genetic disorder that arises due to mutations in the Cystic Fibrosis Transmembrane Conductance Regulator (*CFTR*) gene, which produces a protein responsible for epithelial ion transport. This leads to a disruption in transepithelial Cl-, Na + and HCO_3_− ion transport and the subsequent dehydration of the epithelium within a range of organs including the respiratory system, pancreas, reproductive system, and sweat glands [1, 2]. Foremost is the disruption to the respiratory tract, which becomes enveloped with thickened mucus due to an osmotically-driven reduction in airway surface liquid volume, thus reducing mucociliary clearance [2, 3], and facilitating colonisation by pathogenic organisms. This leads to a cycle of inflammation and infection as pathogens such as bacteria, viruses, and fungi proliferate in the thickened mucus of the conducting airways. The cycle of infection and inflammation leads to fibrosis of the airways, pulmonary insufficiency and bronchiectasis which together ultimately leads to respiratory failure. Importantly, while there is a failure to clear thickened mucus there also appears to be a lack of, or dysfunction in, an auto-feedback mechanism preventing goblet cells from continually over-producing mucins, leading to mucus plugging and mucus plaques [4].

Fibrosis in other lung diseases including asthma, chronic obstructive pulmonary disease (COPD) and idiopathic pulmonary fibrosis (IPF) has been well characterised. One of the driving forces behind fibrosis is Epithelial Mesenchymal Transition (EMT), a mechanism first identified in the 1980s [5]. EMT is the process of epithelial cells losing epithelial proteins including E-Cadherin, which is responsible for tight junctions [6, 7], and the miRNA200 family which helps maintain an epithelial phenotype [8]. The cell moves to a more mesenchymal phenotype as it gains mesenchymal markers such as N-Cadherin (providing the cells with migration potential) [9], Vimentin and Fibronectin (proteins excreted to help form the extracellular matrix) [7, 10], and the fibroblast proliferation transcription factors Snail, Slug and Twist [7, 11]. Furthermore, upon the loss of the tight junctions and pseudo-stratified phenotype, epithelial cells become flattened and take on a stratified squamous epithelium appearance, allowing them to migrate throughout the tissue [12, 13].

When tissue is damaged/wounded or invaded by foreign antigens such as viruses and bacteria, a series of signaling cascades activate the immune system, resulting in inflammatory responses that lead to EMT [14, 15]. Macrophages, neutrophils, eosinophils and other immune cells are recruited to the damaged tissue and release an array of cytokines and growth factors including transforming growth factor β1 (TGF-β1) that signal the tissue to repair itself [16–19]. A study looking at kidney fibrosis estimated that during the repair process, 35% of fibroblasts that populate the tissue under repair are from epithelial/endothelial mesenchymal transition, 12% are derived from bone marrow (BM) via cell migration through the CXCL12/CXCR4 axis, and 30% are resident cells [15]. However, when tissue is persistently damaged, this leads to chronic inflammation, increased and prolonged EMT, and increased fibroblast proliferation resulting in hyperplasia [20]. Fibroblastic cells become activated to form myofibroblasts that excrete products that create a disorganised extracellular matrix. The accumulation of this matrix leads to permanently damaged fibrotic tissue with an aberrant architecture that is unable to function correctly [21, 22].

Over the last decade, the origin of proliferating fibroblasts during tissue repair has become a focus of a large body of research designed to better understand and prevent tissue fibrosis. The build-up of fibrotic tissue can lead to hepatic cirrhosis, nephrogenic systemic fibrosis and pulmonary fibrosis resulting in ongoing pathology and scarring of the affected organs, leading to early death unless the affected organ is removed and replaced through transplantation [23–25].

EMT is associated with many processes, including embryonic development, wound healing and tissue repair, and cell migration. These processes are subdivided into three distinct EMT categories:

- *Type 1*: During embryonic implantation onto the uterine epithelium, primitive epithelial cells in the trophoectoderm undergo EMT and migrate within the inner cell mass and undergo mesenchymal to epithelial transition (MET) to form cells that will go on to form the various organs [15, 26, 27].
- *Type 2*: Tissue damage occurs over a prolonged period and leads to fibrotic tissue. Despite being termed epithelial to mesenchymal transition; this process is not confined to epithelial cells. Endothelial cells and pericytes have also been observed to undergo a very similar process, indicating that this process is important for more than just epithelial tissue repair [28–30].
- *Type 3*: The largest body of research has been on type 3 EMT which is involved in malignant cell growth leading to metastasis [26, 27]. With the removal of tight junctions through down regulation of E-Cadherin, transdifferentiated epithelial cells are free to migrate to areas of damage through the tissue and even the bloodstream if required. However, this process can be utilised by cancerous cells which, after undergoing EMT, have the potential to metastasize and form secondary tumor growths within distant organs [31]. Once metastases have occurred, prognosis is diminished because the cells can evade treatments such as chemotherapy, making many treatments redundant and potentially more dangerous than beneficial [32, 33]. The remainder of this review will focus on Type 2 EMT, and for convenience will be referred to simply as EMT.

## EMT signaling: A pathway to fibrotic tissue

**Fig. 1:**
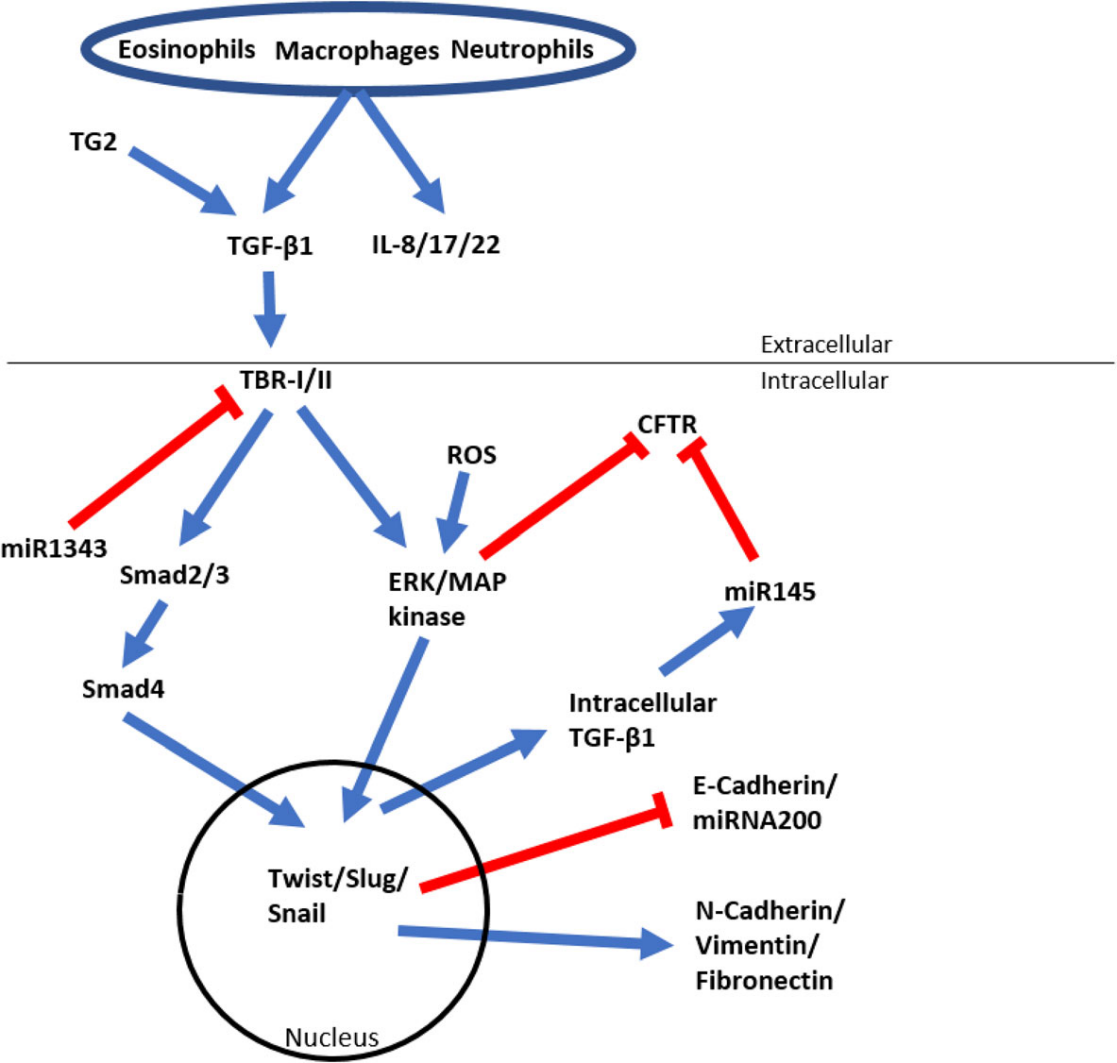
A schematic diagram of the TGF-β1 signaling cascade leading to EMT (blue arrows indicating active signal pathway, red lines indicating inhibition)

EMT is a complex process that involves a large interactome including protein to protein and genetic interactions that are initiated and controlled as a response to extracellular cues. At the forefront of these interactions is TGF-β1 which, on addition to epithelial cultures, causes the cells to undergo EMT (Fig. 1) [7, 10]. TGF-β1 is involved in several cellular functions including cell proliferation, cell differentiation and apoptosis [34–36]. TGF-β1 performs these various actions by directly activating a range of signaling pathways including Smad proteins, ERK/MAP kinases and micro RNAs [37–41], indicating that the progression of EMT is caused by a complex cascade of multiple signal transductions. TGF-β1 binds to target molecules on the cellular membrane known as TGF-β type I and type II receptors (TBR-I and II) creating a cascade of signals [37]. Activation of pathway-restricted Smads form heterodimers with Smad4 and then translocate to the nucleus. Within the nucleus they can promote the transcription of Snail and Twist, which together help break down E-cadherin and subsequently the tight junctions and adherens junctions, resulting in a leaky epithelium [42]. Apical polarity of the cells is then lost and cells are able to detach from the basal lamina, allowing them to break free of the tissue and become mobile [43–45]. A range of EMT inducers act directly or indirectly with TGF-β1, or via alternative pathways including reactive oxygen species (ROS) that are generated through hypoxic conditions, Fibroblast Growth Factor-2 (FGF-2), Epidermal Growth Factor (EGF), Connective Tissue Growth Factor (CTGF) and Transglutaminase 2 (TG2) which can activate matrix bound TGF-β1 [46–49].

## EMT, an underlying role in fibrotic lung diseases

Kim et al. reported that terminally-differentiated airway epithelial cells (AECs) could transdifferentiate into fibroblasts and myofibroblasts and develop fibrotic tissue [50]. Using a mouse model expressing β-galactosidase exclusively in lung epithelial cells, they tracked the epithelial cells under pulmonary fibrotic conditions and found β-galactosidase positive cells also expressing the mesenchymal markers α-smooth muscle actin (α-SMA) and Vimentin, showing that lung epithelial cells can transdifferentiate into other cell types. Subsequently it was shown that EMT in various lung diseases is the underlying cause of fibroblast, goblet cell and pneumocyte hyperplasia, which leads to lung fibrosis [51, 52]. The following sections outline and demonstrate the role of EMT in asthma, IPF, viral infections, COPD, and why it is important to gain more insight into its role in cystic fibrosis.

### Asthma

Asthma is an inflammation and swelling of the airways that results in airway narrowing, goblet cell hyperplasia and airway hyper-responsiveness that make it difficult to breathe [51]. Severity can range from mild to severe. Human air liquid interface cultures (ALI) cultures developed from the epithelial cells of normal and asthmatic patients have been examined for their response to TGF-β1 exposure. While E-cadherin expression was initially lower in the asthmatic ALIs, treatment with TGF-β resulted in significantly decreased E-cadherin expression, whereas expression in normal ALIs did not decrease significantly [10].

Fibronectin, which was absent without TGF-β1 exposure in both asthmatic and normal ALIs, was then expressed after TGF-β1 addition, but at a much greater level in asthma ALIs [10]. Furthermore, histological analysis of the ALI cultures revealed that markers of EMT were far more extensive throughout the epithelial layers in asthma ALIs compared to normal, and in the latter those markers were primarily localised to the basal epithelial layer. Since tight junctions within the asthmatic epithelial airways are often disturbed, TGF-β1 released from immune cells might be able to access and affect cell layers deeper into the epithelium [42, 53]. The varying severity of asthma also appears to alter epithelial cell responses to TGF-β1, with Johnson et al.*,* [9] showing that TGF-β1 had a greater effect on cells obtained from severe asthmatic subjects [9]. A range of markers including *EFNB2, FGFR1, FGFR2, INSR, IRS2, NOTCH2, TLE1, NTRK2 and ADAM33* are all dysregulated in asthma patients [54, 55], and IL-22, a pro-inflammatory cytokine produced by immune cells, is significantly elevated in severe asthmatic subjects compared to mild asthmatic subjects [9]. Together these studies show that EMT is associated with asthma severity, but whether modifying the EMT response has therapeutic applications remains to be seen.

### Idiopathic pulmonary fibrosis

Idiopathic Pulmonary Fibrosis (IPF) is a distinct variety of progressive fibrosing interstitial pneumonia associated with declining lung function, and is caused by increasing amounts of fibrotic tissue that cannot be correctly repaired by the lung [56]. To date, IPF is irreversible and has a 5-year survival of 43% [57, 58]. Interleukin-17 (IL-17) can induce *TGF-β1* gene and protein expression in IPF animal models and IPF patients through the Smad2/3 and ERK1/2 pathways [59], and blocking TGF-β1 in rat models has been shown to slow disease progression [60]. Immunohistological analysis of human IPF patients has shown increased cell proliferation to repair the tissue through increased cytokeratin 14 (CK14) expression, a marker for airway basal progenitor cells. E-Cadherin expression extends into the basal cells as well as deeper into the underlying tissue and appears to be colocalised with the N-Cadherin expression associated with mesenchymal cells, indicating that cells throughout the epithelium are in the process of transition from epithelial cells to mesenchymal cells [13]. Recently, tannic acid and triptolide has been identified as a potential drug to slow IPF, through binding to the active site of TGF-β1 [61, 62]. In vitro results have shown that following tannic acid treatment, TGF-β1 induced Smad2 and Smad3 phosphorylation is diminished, reversing morphogenic and genetic changes in epithelial cell cultures [62]. Other drugs have also recently been examined, including thalidomide (inhibiting the Smad independent pathway), Pirfenidone (inhibits myofibroblast differentiation through mitophagy induction leading to reduced ROS and PDGFR-PI3K and Akt activation) and Tubastatin (Inhibits HDAC6 activated TGF-β1-PI3K-AKT signaling leading to decreased collagen type 1 expression) [63–65]. These results suggest that despite the extensive distribution of EMT-induced fibrosis throughout IPF lungs, retarding fibrosis may be a possibility with the use of drugs that inhibit EMT.

### Viral infections

Viral infections of the airways elicit immune responses leading to tissue repair, and they have also been found to induce EMT in vitro. Specific cell surface binding proteins such as the Epstein-Barr virus (EBV) encoded latent membrane protein 1 (LMP1) in kidneys have been found to directly initiate EMT [66], as do secondary infections such as enterotoxigenic *Escherichia coli* in intestinal epithelial cells [67]. Human cytomegalovirus (HCMV), human papillomavirus (HPV), hepatitis C virus (HCV) and respiratory syncytial virus (RSV) have been shown to induce morphological changes, switch from epithelial to mesenchymal markers, and increase proliferation and migration of non-invasive cancer cell lines [68–71]. Repeated rhinovirus infections as a child can also increase the likelihood of developing asthma [72, 73]. Minor, et al. showed in vitro that the addition of rhinovirus (RV) was sufficient to induce EMT, although the effect was significantly enhanced with the addition of TGF-β1 [74]. This effect may be due to TGF-β1 mediated silencing of the protective mucosal interferon (IFN)-I and III production through the down-regulation of inducible interferon regulatory factor 1 (IRF1) expression in mesenchymal cells, which has been shown to increase both RV and RSV replication [75]. Interestingly, the measles virus, which can infect polarised epithelial cells, is unable to infect epithelial cells after they have undergone EMT, likely because the measles virus cellular receptor (which is still unknown) is down-regulated during EMT [76]. Other viruses that have been shown to induce EMT include the human immunodeficiency virus (HIV) through the hedgehog pathway [77], and Transmissible gastroenteritis virus (TGEV) through the TGF-β/PI3K/ERK pathways [67].

Whether EMT results from a viral infection, or EMT provides a suitable environment for viral infection are both important notions to consider as they can each have ramifications for diseases that result in organ fibrosis such as cystic fibrosis. Identifying which of these two paths is the primary cause will allow us to understand the fibrotic process more deeply and provide ways of controlling it, as well as preventing secondary infections.

### Chronic obstructive pulmonary disease

Chronic Obstructive Pulmonary Disease (COPD) results from chronic inflammation, pulmonary remodeling, permanent airflow obstruction, and air trapping that leads to difficulties breathing [78, 79]. EMT is present in COPD, and in patients with COPD who are still smoking, the leaky epithelium, goblet cell hyperplasia, and poorly formed architecture of the airways results in more prevalent EMT [80–83]. Milara et al., (2013) showed in vitro and in vivo that E-cadherin was almost absent in both smokers and COPD patients, while collagen type 1 and Vimentin expression was far more prevalent compared to non-smokers [84]. From airway biopsies of COPD patients, expression of TGF-β1 and its downstream signaling partners Smad2/3 were greatly increased in COPD patients compared to normal, with the most prominent expression around blood vessels [38]. Mahmood et al., [85] found that there was a distinct difference between the small and large airways, with Type 2 EMT primarily found within the small airways leading to fibrotic tissue, and Type 3 EMT more closely associated with large airways leading to COPD-related cancers [85].

The canonical Wnt signaling pathway in COPD/EMT has also been shown to be up-regulated, as indicated by cellular-compartment expression of β-catenin in epithelial cells that is positively correlated with the EMT markers Twist and Snail [80]. Amongst current smokers with and without COPD, there was a shift from cytoplasmic to nuclear staining for β-catenin, Twist and Snail in basal cells, reticular basement membrane cells and lamina propria cells.

ALI cultures using COPD cell lines that are simply maintained and not exposed to cigarette smoke show that mesenchymal markers present early in the cultures are lost over time, indicating that EMT-related fibrosis can be halted in vitro, provided that the relevant EMT-causing stimuli (i.e. cigarette smoking) are removed [83]. Elevated heparin-binding epidermal growth factor (HB-EGF) has recently been linked to COPD disease severity by increasing EMT and collagen deposition [86]. However, the use of inhaled corticosteroids can decrease EMT activity in COPD patients through a reduction in epidermal growth factor receptor (EGFR) expression, suggesting a potential method for slowing or halting the development of COPD [87].

Unlike the lung diseases discussed earlier, it seems that EMT related to COPD is a direct result of cigarette smoke, and the elimination of this stimulus can slow down the progression of EMT induced fibrosis.

## Cystic fibrosis

Research examining the involvement of EMT in CF has been limited, focusing only on CFTR involvement in cancer and other fibrotic diseases [88–90]. If new respiratory therapeutics extend the life expectancy of CF patients by 20+ years then the CFTR deficiency in other organs may have more severe effects on life and functioning, with CF patients already at a 17× higher risk of developing gut cancers [91]. The mechanisms of this interaction with cancers remain unknown, and what effects these processes will have should be elucidated well before they become a problem.

### Linking CFTR with EMT

*Pseudomonas aeruginosa* infection (a key and common infection in CF patients that results in production of an excess of largely ineffective neutrophils) can induce TGF-β1 driven EMT by activating monocytes [92]. TGF-β1 is a known CF modifier gene that can influence the severity of respiratory CF disease based on *TGF-β1* polymorphisms as well as environmental factors such as smoking which exacerbate or reduce respiratory severity by modulating TGF-β1 signaling [93–95].

Recently, an increase in UDP-glucose levels (an extracellular nucleotide that helps regulate mucociliary clearance) in CF lung secretions was shown to recruit neutrophils through the upregulation of interleukins [96]. Neutrophils have been shown to excrete neutrophil-derived elastase which can cleave E-Cadherin [97]. The epithelial hyperplasia present in the airways of CF mice, where a 5-fold increase in basal epithelial cells with clonogenic/proliferative potential has been reported [98], indicates that CF lungs undergo increased tissue remodeling and repair, consistent with an EMT process.

A portion of airway basal cells are progenitor cells that can self-renew and differentiate through two basal cell sub-types; basal stem cells and basal luminal progenitors. After epithelial injury the basal luminal progenitor cells become either ciliated cells or mucin secretory cells [99, 100]. How these cells divide and expand throughout the injured epithelium to repair the wound is still largely unknown, but it is possible that basal cells in these circumstances undergo at least a partial EMT process. This clonal expansion may not just be an inflammatory response, but potentially driven by the CFTR deficiency itself. Recently, TG2 (an EMT inducer that works through TGF-β1) was found to be elevated in vitro in CF epithelial cell cultures leading to increased TGF-β1 and EMT induction. Inhibition of TG2 could reverse the EMT process, lower TGF-β1 gene expression, reduce the amount of extracellular matrix bound TGF-β1 and stabilise CFTR [49].

The close association of EMT (Particularly type-3 EMT which leads to metastasis) with cancer led to the identification that CFTR is often down-regulated in metastatic cancer cells [88, 101]. Maloney et al., (2016) showed higher levels of circulating TGF-β1 in CF patients [102]. TGF-β1 decreases CFTR expression through the p38 MAPK pathway and interestingly this was shown to occur prior the classical EMT E-Cadherin to N-Cadherin shift with low TGF-β1 concentrations [103]. E-Cadherin/N-Cadherin co-localisation along with CFTR downregulation throughout ALI cultures treated with TGF-β1 has been reported and suggested that this is evidence that EMT is not occurring [104]. However, this phenomenon was also shown by Jonsdottir et al. who suggest that this may just be an intermediate phase in the EMT process [13].

The recent insights into the roles of micro RNAs have shown that they are key factors in both CFTR regulation as well as EMT with miR1343 binding to the 3’UTR of TGFRβ 1 and 2, resulting in unstable mRNA transcripts thus reducing the level of TGF-β1 signaling while miR145 which is upregulated due to TGF-β1 binds to the 3’UTR of CFTR causing reduced CFTR expression [41, 105].

The addition of TGF-β1 to non-invasive breast cancer cells caused the cells to undergo type-3 EMT as seen by the decrease in E-cadherin, but interestingly CFTR was also down-regulated [88]. To determine whether CFTR down-regulation was connected with E-cadherin down-regulation and EMT, rather than just a side effect of TGF-β1 addition, the non-invasive cells were treated with a CFTR inhibitor, resulting in a decrease in E-cadherin expression [101]. When a metastatic cancer cell line was made to over-express CFTR, upon subcutaneous injection into mice a reduced number of metastatic lung growths resulted compared to the same cell line without CFTR over-expression [88].

The direct implication of CFTR in cancer progression is still somewhat controversial since CFTR has not yet been connected in any direct signaling pathways, however, CFTR may act by regulating intracellular Cl^−^ concentrations [106], and so influencing the intracellular environment.

*C-Src* (a tyrosine kinase) has also been linked to EMT [107, 108], and also found to be regulated by CFTR. Although CFTR normally suppresses the oncogene *c-Src*, when CFTR is impaired *c-Src* is up-regulated [109]. *c-Src* is highly expressed in 60% of cancers and is involved in cell proliferation, cell survival, angiogenesis and invasion pathways [110]. The transcription factor NFkB is activated by c*-Src* which in turn up-regulates genes such as *MUC1,* a glycoprotein normally present in lung mucus and is required for mucociliary clearance, but is also highly secreted in CF causing increased mucus to build up and creating an environment for bacterial infection [111]. As a result, in a paracrine fashion *c-Src* could affect cells that don’t normally express CFTR. *c-Src* levels in cells from *CFTR* knockout animal cell lines can be returned to normal with the addition of an IL-1β inhibitor [112].

CFTR is down regulated in COPD patients [113, 114], and cigarette smoke has been identified as a possible initial cause of this down regulation through a rise in cytoplasmic Ca^2+^ which potentially prevents normal sorting/degradation of CFTR, and results in the rerouting of the CFTR protein from cellular membrane to aggresomes. Chelation of Ca^2+^ prevented this rerouting and maintained normal CFTR activity on the cellular membrane [90].

Whether CFTR/c-Src/MUC1 interaction is directly involved in the development of hyperplasia and the increased number of stem cells in CF lungs is not known, but significant therapeutic possibilities warrant investigating the role of both type-2 and type-3 EMT in CF lung disease. Ultimately, if EMT is linked to CFTR dysregulation, then using methods to block EMT, such as small molecule drugs like Kaempferol and TGF-β1 receptor kinase inhibitors may assist in reducing both hyperplasia and lung fibrosis [115, 116].

## EMT increases cell plasticity

EMT is a complex physiological response process that occurs when tissues are damaged. There is increasing evidence that the traditional concept that once cells had terminally differentiated they would carry out their function before dying and being replaced by a progenitor cell that differentiates into the required cell type, is outdated. It now appears that ‘terminally differentiated’ cells may in fact be a source of these progenitor cells, and indeed Cre lineage tracing experiments in mice show that Club cells can act as a source of progenitor cells for the ciliated cells of the lung [117]. The inhibition of TGF-β1 after EMT induction does result in a transition of the mesenchymal cells back into epithelial cells [10], however the stem cell potential of these de-differentiated epithelial cells may be far wider than the original epithelial cell type. Battula et al., reported that once epithelial cells had undergone EMT in vitro, they were then capable of differentiating down the osteogenic, adipogenic and chondrogenic lineages. These de-differentiated epithelial cells also expressed markers that are associated with mesenchymal stem cells (MSC), while maintaining some markers of epithelial cells [118]. The residence of MSCs within organs is unknown, raising the question; are MSCs a niche population of cells within tissues, or are they various cell types that can transdifferentiate into other cell types. In bone marrow MSCs, various populations of cells have markers that are associated with specific cell lineages, resulting in them preferentially differentiating down those cell lineages [119, 120]. The capability for preferential differentiation is well established amongst MSC isolates from different tissues such as dental pulp, which preferentially differentiate down the odontogenic and neurogenic pathway [121]. A micro-array of human mammary epithelial cells that have been induced to undergo EMT were compared to bone marrow isolated MSCs showed 70% similarity in expressed genes, with 15% of the differentially regulated genes being epithelial markers left from prior to EMT induction [118].

Therefore, if MSCs are indeed de-differentiated cells that were once thought to be terminally-differentiated, this change in viewpoint may allow us to approach controlling the EMT process, to manipulate and direct tissue regeneration, particularly in CF patients where diagnosis is typically neonatal and well before fibrosis of tissue begins to occur.

## Conclusion

In conclusion, EMT is a universal and normal process involved in tissue repair, but EMT dysregulation can lead to fibrosis. These processes have already been well studied in a range of lung diseases. While EMT is less understood in the pathology of CF, studies do show the involvement of CFTR in the EMT process, particularly in Type 3 EMT, where there has been compelling evidence of its involvement in cancer progression in lung, gut, liver and breast cancer. Type 2 EMT involvement in CF should be explored further to understand the process in the airways of CF patients as the results may provide novel insights into the causes and effects of the dysregulated cellular pathways, potentially providing a future means for preventing or limiting CF related airway disease. As treatments for CF lung disease improve, an improved understanding of EMT in CF lung disease may also benefit our understanding of CF disease in other organs. While there are several strategies being developed to slow or halt CF lung disease [122], where that disease already exists there remains a need to restore tissue architecture through resolution of the fibrotic response. A fuller understanding of the mechanisms behind EMT and lung fibrosis may allow us to prevent, halt or even reverse the fibrosis process.

## Abbreviations

ALI: Air Liquid Interface
BM: Bone Marrow
CF: Cystic Fibrosis
*CFTR*: Cystic Fibrosis Transmembrane Conductance Regulator
COPD: Chronic Obstructive Pulmonary Disease
EMT: Epithelial Mesenchymal Transition
IPF: Idiopathic Pulmonary Fibrosis
MET: Mesenchymal Epithelial Transition
TGF-β1: Transforming Growth Factor-β1
α-SMA: α-Smooth Muscle Actin
